# Life-Threatening Anaphylaxis Following Intravenous Ondansetron in a Previously Healthy Woman: A Case Report

**DOI:** 10.7759/cureus.94355

**Published:** 2025-10-11

**Authors:** Md. Aziz Mazumdar, Sonali Bansal, Kaneez Fatima, Rajat Bhardawaj, Ria Midha

**Affiliations:** 1 Medicine, Park Hospital, Gurugram, IND; 2 Critical Care Medicine, Park Hospital, Gurugram, IND; 3 Internal Medicine, Nishtar Medical University, Multan, PAK; 4 Anaesthesia, Park Hospital, Gurugram, IND; 5 Internal Medicine, Park Hospital, Gurugram, IND

**Keywords:** adverse drug reaction, allergy and anaphylaxis, drug-induced hypersensitivity, emergency medicine, ondansetron

## Abstract

Ondansetron is a drug that is prescribed to treat and prevent nausea and vomiting induced by chemotherapy, radiation therapy, surgical procedures, analgesics, or migraine. It is also commonly used in clinical practice for the symptomatic management of nausea and vomiting due to acute gastroenteritis.

The drug is generally acknowledged for its safety, apart from common side effects such as constipation, hot flushes, and headaches. Some rare adverse events, including arrhythmias and QT interval prolongation, have been reported in isolated cases in the literature. However, severe anaphylactic reactions are rarely reported but hold significant value.

We describe a rare case of potentially fatal anaphylaxis to a commonly used and generally considered safe medicine. The patient, a 56-year-old female with no known comorbidities, arrived at the Emergency Room complaining of nausea and recurrent vomiting, possibly due to the ingestion of unhygienic, undercooked food. She was hemodynamically stable, with signs of dehydration present. Intravenous ondansetron 4 mg and intravenous fluids (Ringer's Lactate) were administered. Consequently, she developed a sudden severe anaphylactic shock leading to hypotension, tachycardia, and bronchospasm, leading to respiratory distress, managed with endotracheal intubation, vasopressor support, intramuscular adrenaline, and urgent ICU admission. After six hours of intensive treatment, she survived and was extubated uneventfully. Further blood and skin allergy testing confirmed hypersensitivity to ondansetron.

Our case report underscores that rare, severe anaphylactic reactions may occur with drugs typically considered safe, emphasizing early recognition and intervention. Ondansetron is one such medication that is available over the counter in India and other countries, which raises safety concerns. We describe a rare case of potentially fatal anaphylaxis following intravenous ondansetron administration in a previously healthy woman. This case highlights the importance of early recognition and intervention in severe hypersensitivity reactions to commonly used drugs.

## Introduction

Ondansetron is a selective serotonin (5-HT₃) receptor antagonist that blocks receptors located both in the gastrointestinal tract and the central nervous system. It is used to prevent nausea and vomiting induced by chemotherapy, radiotherapy, and surgical procedures, and is also prescribed for migraine-related vomiting [1].

Ondansetron is also commonly used in clinical practice for the symptomatic management of nausea and vomiting due to acute gastroenteritis [2].

Ondansetron is associated with several adverse effects, the most common of which include headache, fatigue, malaise, and constipation. More serious reactions may rarely occur, such as QT interval prolongation and severe hypersensitivity responses [3]. Although hypersensitivity to ondansetron is rare, it has been reported [4]. According to recent pharmacovigilance data, the estimated incidence of ondansetron-induced anaphylaxis is less than 1 in 100,000 exposures.

Serious adverse drug reactions are uncommon; however, ondansetron can occasionally cause life-threatening anaphylaxis or anaphylactic shock [5]. Hence, careful administration of the drug and immediate treatment of anaphylaxis is crucial to prevent fatal outcomes.

## Case presentation

A 56-year-old postmenopausal woman presented to the Emergency Department with complaints of diarrhea for five days and persistent vomiting for one day after consumption of undercooked street food. There was no association of diarrhea with fever, rash, or blood in stool; vomiting was non-bilious, non-projectile, and contained food particles. Her medical history was unremarkable for any known comorbidities. On physical examination, she was conscious, afebrile, and well-oriented. The vitals showed hemodynamic stability with a pulse rate of 110/minute, blood pressure of 130/80 mmHg, and saturated oxygen (SPO2) of 98% on room air. On chest auscultation, bilateral air entry was present. She had positive signs of dehydration, such as dry tongue and dry skin, while systemic examination revealed no significant findings.

In the Emergency Room, a peripheral intravenous cannula of 18 G was secured in the right dorsum of the hand. Intravenous ondansetron 4 mg was administered. Within five minutes, the patient became restless and developed skin flushing, respiratory distress, and bronchospasm. Her blood pressure dropped to 80/40 mmHg, oxygen saturation dropped to 74% and sustained sinus tachycardia at 176/minute was noted. On auscultation, bilateral wheeze was present; however, sensorium remained intact.

An anaphylactic reaction to ondansetron was suspected. Immediate resuscitation was initiated with intravenous fluids (Ringer’s Lactate) and vasopressor support. An intramuscular dose of adrenaline (0.5 mg, 1:1000) was administered first. Because hypotension and bronchospasm persisted, an intravenous adrenaline infusion was subsequently started, followed by hydrocortisone 100 mg administered intravenously. In view of persistent respiratory distress and hypotension, endotracheal intubation was done with a cuﬀed oral endotracheal tube sized 7.5 mm under rapid sequence induction.

**Figure 1:**
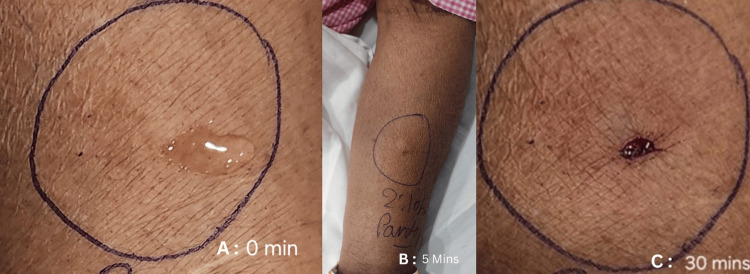
Skin prick test with ondansetron at 0 min, 5 mins, and 30 mins Serial images demonstrating a progressive hypersensitivity response to ondansetron. (A) At 0 minutes: baseline appearance at the intradermal injection site. (B) At 5 minutes: development of a visible wheal (~10 mm) with surrounding flare, although the erythema is subtle in the photograph. (C) At 30 minutes: persistent erythematous wheal (~20 mm), confirming hypersensitivity.

The patient was stabilized and transferred to the Intensive Care Unit for further management. The bedside 2-D Echo showed an ejection fraction of 60% with no regional wall motion abnormality. The patient was managed in the ICU on grounds of anaphylactic shock, and vasopressor support was gradually tapered oﬀ over a period of six hours, and she was extubated uneventfully. Post-extubation, the patient was conscious, oriented, afebrile, and normotensive. Later,a skin prick test for ondansetron was performed, which revealed a positive reaction with a wheal diameter of 20 mm (Figure 1).

Further blood tests demonstrated an elevated serum tryptase level of 45 µg/L (reference range < 11.4 µg/L), confirming ondansetron hypersensitivity [6]. Laboratory investigations are summarized in Table 1.

**Table 1:** Laboratory findings on admission Laboratory parameters of the patient at presentation. All values were within normal limits except for serum tryptase, which was elevated (45 µg/L; reference <11.4 µg/L), confirming mast cell activation consistent with anaphylaxis. ALT: alanine transaminase

Parameter	Patient Value	Reference Range
Hemoglobin	12.8 g/dL	12–16 g/dL
WBC count	7.6 ×10⁹/L	4–11 ×10⁹/L
Platelet count	250 ×10⁹/L	150–400 ×10⁹/L
Sodium	138 mmol/L	135–145 mmol/L
Potassium	4.1 mmol/L	3.5–5.0 mmol/L
Creatinine	0.9 mg/dL	0.6–1.2 mg/dL
ALT	28 U/L	<40 U/L
Serum Tryptase	45 µg/L	<11.4 µg/L

## Discussion

Our discussion aims to highlight the areas of insufficient awareness of the safety profile of ondansetron, insufficient knowledge about the underlying mechanisms of hypersensitivity, and failed responses to initial bolus doses of adrenaline, necessitating further research regarding underlying mechanisms and appropriate treatment strategies for such hypersensitivity reactions.

After a detailed review of the literature, several case reports and case series describing anaphylactic reactions caused by ondansetron are summarized in Table 2.

**Table 2:** Reported cases of ondansetron-induced anaphylaxis Summary of published case reports and case series describing ondansetron-induced anaphylaxis. Data include patient demographics, indication for ondansetron use, type of hypersensitivity reaction, outcome, and remarks. Sources: [7-12] PCOS: polycystic ovary syndrome; AML: acute myeloid leukemia; T-ALL: T-cell acute lymphoblastic leukemia; IgE: immunoglobulin E

Year	Sex	Age	Primary Disease/Indication	Reaction	Outcome	Remarks
2008 [7]	F	19	PCOS/gastritis, IV ondansetron	Urticaria, anaphylaxis	Survived	Off-label use; adrenaline needed
2009 [8]	F	44	Anesthesia induction (ondansetron + others)	Hypotension, raised tryptase	Survived	Skin test positive only for ondansetron
2012 [9]	F	12	Vomiting, ondansetron wafer	Facial edema, biphasic anaphylaxis	Survived	3 doses of adrenaline
2021 [10]	F	82	Recurrent vomiting	Shock, failed therapy	Death	Cardiac arrest despite support
2023 [11]	M	70	General anaesthesia	Shock	Survived	Histamine ↑, tryptase normal → non-IgE pathway
2025 [12]	F	34–40s	AML/T-ALL chemo	Delayed anaphylaxis	2 deaths, 1 survived	Reactions after multiple doses
Our case	F	56	Gastroenteritis, IV ondansetron	Fulminant anaphylaxis	Survived	Required intubation + ICU

Recent case reports by Sapkota and Bhagat (2021) and Suzuki et al. (2023) describe similar episodes of ondansetron-induced anaphylaxis with comparable clinical outcomes [9,10]. To the best of our knowledge, only three case reports exist in the literature where ondansetron led to an anaphylactic shock.

Sapkota and Bhagat presented a case that raised the question of whether a single dose of intramuscular adrenaline is sufficient to treat an anaphylactic reaction to ondansetron. This case described an 82-year-old woman with a background of osteoarthritis treated with 4 mg intravenous ondansetron for repeated episodes of vomiting. She developed an anaphylactic reaction with a failed response to initial intramuscular adrenaline injection, intravenous hydrocortisone, and intravenous pheniramine. Endotracheal intubation was done, and continuous adrenaline infusion was started. Hypotension and respiratory distress failed to reverse, and, unfortunately, the patient died of cardiac arrest despite 30 minutes of attempted resuscitation [9].

Our case also showed a failure of resolution of respiratory distress and hypotension with an initial intramuscular dose of adrenaline. The limited response to intramuscular adrenaline may have been due to reduced absorption from peripheral vasoconstriction during shock, as reported previously. Our patient required adrenaline infusion, endotracheal intubation, and ICU-based care. We would like to highlight the importance of hospital-based care and the availability of treatment escalation for the treatment of anaphylactic reactions to ondansetron. This also raises a question regarding the safety of ondansetron being given in the community and the necessity of the availability of hospital-based treatment facilities if such anaphylactic reactions were to arise.

Another study highlighted the underlying potential mechanisms of hypersensitivity. A 70-year-old man, during induction of general anesthesia, developed an anaphylactic reaction five minutes post-ondansetron administration. He was treated with 5 mg intravenous chlorphenamine instead of the usual intramuscular adrenaline. The patient's blood pressure rapidly improved within a few minutes. Later testing revealed a negative serum tryptase level; however, a raised serum histamine level of 17.2 ng/ml was seen (normal range being 0.5_1.23 ng/ ml). This case adds weight to the mass-related G protein-coupled receptors X2 (MRGPRX2) mediated pathway and the potential role of immediate antihistaminic treatment [10]. The MRGPRX2 receptor on mast cells mediates non-IgE-dependent degranulation, explaining reactions with elevated histamine but normal tryptase levels, such as in similar ondansetron cases.

We, therefore, urge that further research is needed to assess the safety profile of ondansetron and to evaluate underlying biochemical pathways and appropriate treatment protocols for such hypersensitivity reactions. Although specific preventive strategies are not well-established, slow intravenous administration (over 2-5 minutes) and close monitoring are recommended to reduce the likelihood of acute hypersensitivity.

## Conclusions

Owing to its well-established safety and efficacy, ondansetron has become a commonly prescribed drug in the management of nausea and vomiting. Although hypersensitivity reactions are rare, this drug can lead to life-threatening anaphylactic reactions on first and repeated exposure, which is alarming for clinicians. The risk is particularly concerning in resource-limited settings. Importantly, in India and several other countries, ondansetron is available as an over-the-counter drug, raising the danger of unsupervised use outside the hospital. Public education, cautious prescribing, and preparedness for immediate resuscitation are essential. Clinicians should remain vigilant and prepared for prompt management when prescribing this commonly used drug.
